# Different patterns of cerebral perfusion in SLE patients with and without neuropsychiatric manifestations

**DOI:** 10.1002/hbm.24837

**Published:** 2019-10-24

**Authors:** Zhizheng Zhuo, Li Su, Yunyun Duan, Jing Huang, Xiaolu Qiu, Sven Haller, Haiyun Li, Xiaofeng Zeng, Yaou Liu

**Affiliations:** ^1^ Department of Radiology Beijing Tiantan Hospital, Capital Medical University Beijing China; ^2^ School of Biomedical Engineering Capital Medical University Beijing China; ^3^ Department of Rheumatology Peking Union Medical College Hospital, Peking Union Medical College & Chinese Academy of Medical Science Beijing China; ^4^ Key Laboratory of Rheumatology and Clinical Immunology Ministry of Education, National Clinical Research Center on Rheumatology, Ministry of Science & Technology Beijing China; ^5^ Department of Imaging and Medical Informatics University Hospitals of Geneva and Faculty of Medicine of the University of Geneva Geneva Switzerland

**Keywords:** 3D arterial spin labeling, neuropsychiatric systemic lupus erythematosus, perfusion, systemic lupus erythematosus

## Abstract

To investigate brain perfusion patterns in systemic lupus erythematosus (SLE) patients with and without neuropsychiatric systemic lupus erythematosus (NPSLE and non‐NPSLE, respectively) and to identify biomarkers for the diagnosis of NPSLE using noninvasive three‐dimensional (3D) arterial spin labeling (ASL). Thirty‐one NPSLE and 24 non‐NPSLE patients and 32 age‐ and sex‐matched normal controls (NCs) were recruited. Three‐dimensional ASL‐MRI was applied to quantify cerebral perfusion. Whole brain, gray (GM) and white matter (WM), and voxel‐based analysis (VBA) were performed to explore perfusion characteristics. Correlation analysis was performed to find the relationship between the perfusion measures, lesion volumes, and clinical variables. Receiver operating characteristic (ROC) analysis and support vector machine (SVM) classification were applied to differentiate NPSLE patients from non‐NPSLE patients and healthy controls. Compared to NCs, NPSLE patients showed increased cerebral blood flow (CBF) within WM but decreased CBF within GM, while non‐NPSLE patients showed increased CBF within both GM and WM. Compared to non‐NPSLE patients, NPSLE patients showed significantly reduced CBF in the frontal gyrus, cerebellum, and corpus callosum. CBF within several brain regions such as cingulate and corpus callosum showed significant correlations with the Systemic Lupus Erythematosus Disease Activity Index (SLEDAI) and the Systemic Lupus International Collaborating Clinics (SLICC) damage index scores. ROC analysis showed moderate performance in distinguishing NPSLE from non‐NPSLE patients with AUCs > 0.7, while SVM analysis demonstrated that CBF within the corpus callosum achieved an accuracy of 83.6% in distinguishing NPSLE from non‐NPSLE patients. Different brain perfusion patterns were observed between NPSLE and non‐NPSLE patients. CBF measured by noninvasive 3D ASL could be a useful biomarker for the diagnosis and disease monitoring of NPSLE and non‐NPSLE patients.

## 1 INTRODUCTION

Neuropsychiatric systemic lupus erythematosus (NPSLE) is a severe and life‐threatening type of SLE. It has a wide range of clinical neurological and psychiatric manifestations (e.g., stroke, epilepsy, cognitive deficits, psychosis, and mood disorders) that decrease quality of life (Ercan et al., 2015; Rhiannon, 2008). Complex underlying pathological mechanisms are reported in SLE, particularly in NPSLE, including neuroinflammation, demyelination, neuron injury, and vasculopathy (Sibbitt et al., 2010). It is crucial to differentiate NPSLE from SLE without neurologic or psychiatric symptoms (named non‐NPSLE; Sarbu et al., 2015). However, the discrimination of NPSLE from non‐NPSLE is difficult in a clinical setting, requiring stable and reliable biological and radiographical markers (Castellino, Govoni, Giacuzzo, & Trotta, 2008; Cervera et al., 2006; Sarbu, Bargalló, & Cervera, 2015).

Perfusion imaging has been used to assess the microvasculature changes in SLE patients. The cerebral hypoperfusion of frontal, temporal, and partial areas has been observed in NPSLE patients by positron emission tomography (PET) and single‐photon emission computed tomography (SPECT) studies, which could provide early biomarkers for SLE (Kodama et al., 1995; Sahebari, Rezaieyazdi, Khodashahi, Abbasi, & Ayatollahi, 2018). Recently, MR perfusion imaging studies with dynamic susceptibility contrast (DSC) reported that the cerebral blood volume (CBV), and cerebral blood flow (CBF) of certain specific brain regions (e.g., posterior cingulate gyrus) were highly related to NPSLE (Gasparovic et al., 2010; Papadaki et al., 2018; Wang et al., 2012; Zimny et al., 2014). Compared to normal controls (NC), both hyperperfusion and hypoperfusion, were illustrated within gray (GM) and white matter (WM) in SLE patients (Gasparovic et al., 2010; Papadaki et al., 2018; Wang et al., 2012; Zimny et al., 2014). Previous studies have demonstrated hypoperfusion within GM and WM in NPSLE compared to non‐NPSLE (Papadaki et al., 2018; Wang et al., 2012). Accumulated evidence indicates that abnormal perfusion (CBF or CBV) is not specific to NPSLE because perfusion is also altered in non‐NPSLE patients. Discordant results were shown in a few studies (Emmer et al., 2010; Waterloo et al., 2001). Emmer et al. (2010) used ROIs (regions of interest), including subcortical GM and central WM, to investigate the perfusion alterations in NPSLE but did not find any significant difference among groups (NPSLE patients, non‐NPSLE patients and NCs).

In clinical practice, the attribution of neuropsychiatric manifestations to SLE mainly depends on expert physician judgment. Neither clinical measurements nor conventional imaging has adequate diagnostic precision. The abovementioned PET and DSC‐MRI perfusion imaging techniques have high sensitivity (80–100%) but low specificity (<67%) in diagnosing NPSLE (Castellino et al., 2008; Kao et al., 1999; Kao et al., 1999). PET, SPECT, or DSC‐MRI are not suitable for every SLE patient due to irradiation or contrast injection.

Arterial spin labeling (ASL) imaging, which is a contrast agent‐free perfusion imaging technique, has been validated as being useful for the evaluation of brain perfusion related to various neurological and psychiatric diseases, including Alzheimer's disease, Parkinson's disease, brain tumor, stroke, seizures, and schizophrenia (Haller et al., 2016; Le et al., 2014; Oura et al., 2018; Schneider et al., 2019). In particular, the development of three‐dimensional (3D) ASL‐MRI with GRASE (a technique combining gradient rapid echo and spin echo) or FSE (fast spin echo) acquisition dramatically improves the perfusion image quality with a high signal‐to‐noise ratio and spatial‐resolution (Alsop et al., 2015; Uetani et al., 2018). However, no previous investigation has focused on the ability of ASL to diagnose and evaluate brain perfusion in NPSLE patients.

Therefore, the purpose of this study was to investigate the underlying perfusion pattern of NPSLE and non‐NPSLE using noninvasive 3D ASL‐MRI and to evaluate its clinical significance.

## 2 MATERIALS AND METHODS

### 2.1 Subjects

From January 2017 to February 2018, a total of 111 female subjects, including 40 clinically diagnosed NPSLE patients (aged 32.75 ± 12.68 years) and 35 non‐NPSLE patients (aged 29.24 ± 9.84 years) from the Department of Rheumatology, Peking Union Medical College Hospital and 36 female age‐matched NCs (aged 32.50 ± 10.57), were enrolled in this study. SLE and the manifestations of NPSLE were diagnosed and defined according to the American College of Rheumatology (ACR) classification and Hanly B criteria for SLE (Hanly et al., 2010; Singh et al., 2006). The inclusion criteria were as follows: (a) age between 18 and 65 years old; (b) right‐handedness; and (c) no contraindications for MRI. The exclusion criteria were as follows: (a) history of alcohol or drug abuse; (b) history of central nervous system disease, such as Alzheimer's disease, Parkinson's disease or depression; (c) lesions in NCs by MRI examination; (d) secondary NPSLE due to other causes or peripheral NPSLE; and (e) poor MRI image quality. The clinical evaluation of current SLE disease activity index by the Systemic Lupus Erythematosus Disease Activity Index 2000 (SLEDAI‐2K) scores, accumulative disease damage index scores by the Systemic Lupus International Collaborating Clinics (SLICC) classification criteria and other clinical variables (including disease duration, total cumulative duration of steroid use, manifestations of SLE, vascular risk factors, and immunological indicators) were recorded (for details, refer to Table 1 and Table S1). According to the above inclusion criteria, exclusion criteria and completeness of clinical information, 9 NPSLE patients were excluded due to incomplete scanning, history of hemicrania or incomplete disease history; 11 non‐NPSLE patients were excluded due to incomplete scanning, history of hemicrania, or unclear NP history; and 4 NCs were excluded due to incomplete scanning or poor image quality. In total, 31 NPSLE, 24 non‐NPSLE, and 32 NCs were included in the final analysis.

**Table 1:** Demographics, clinical information, and CBF values of the subjects

Parameters	NC (32)	Non‐NPSLE (24)	NPSLE (31)	ANOVA or Kruskal–Wallis H (P)	NC versus non‐NPSLE (P)	NC versus NPSLE (P)	Non‐NPSLE versus NPSLE (P)
Age (years)	32.93 ± 10.75	29.13 ± 10.03	32.36 ± 12.61	0.422^a^	0.182^b^	0.827^b^	0.318^b^
Disease duration(month)	–	28 (2–120)	60 (3–468)	–	–	–	0.090^c^
SLEDAI	–	4 (0–12)	6 (0–43)	–	–	–	0.042^c^
SLICC	–	0 (0–1)	1 (0–4)	–	–	–	<0.001^c^
Total cumulative duration of steroid use (month)	–	22.5 (0–192)	21.5 (0–468)	–	–	–	0.408^c^
WM lesion volumes(ml)	–	6.57 ± 3.08	9.29 ± 1.42	–	–	–	0.469^c^
WB CBF(ml/100 g/min)	31.99 ± 4.32	36.02 ± 13.63	33.65 ± 14.69	0.208^d^	0.446^c^	0.145^c^	0.149^c^
GM CBF(ml/100 g/min)	39.99 ± 5.62	43.98 ± 13.77	39.95 ± 15.00	0.383^a^	0.438^b^	1^b^	0.436^b^
WM CBF(ml/100 g/min)	19.66 ± 2.54	23.76 ± 14.10	23.94 ± 14.82	0.923^d^	0.667^c^	0.967^c^	0.786
CBF ratio of GM/WM	2.03 ± 0.13	1.99 ± 0.29	1.80 ± 0.33	0.004^d^	1^c^	0.002^c^	0.009^c^

*Note*: Data were presented by mean ± standard deviation or median(range). More clinical information can refer to Table S1.

a ANOVA.

b Post hoc Tukey.

c Mann–Whitney U test.

d Kruskal–Wallis H test.

All subjects were informed of the purpose of the collection of their information, and this study was approved by the Human and Animal Ethics Committee of Peking Union Medical College Hospital.

### 2.2 Image acquisition

High‐resolution 3D T1‐weighted and 3D ASL MRI imaging was performed for each subject in a GE 3T MR scanner with a 32‐channel receiving coil (GE MR750, GE Healthcare). The 3D T1w images were obtained by a 3D T1 MPRAGE (Magnetic Prepared RApid Gradient Echo) sequence with the following parameters: TR/TE = 1,600 ms/2.13 ms; inversion time = 1,000 ms; Flip angle = 9°; field of view = 240 mm × 240 mm × 160 mm; acquisition voxel = 1 mm × 1 mm × 1 mm; sense factor 1 for slice encoding direction and 2 for phase encoding direction; and acquisition time = 6 min. The 3D pseudocontinuous ASL images were obtained by a 3D TSE sequence with the following parameters: TR/TE = 4,844 ms/10.5 ms; flip angle = 111°; field of view = 240 mm × 240 mm; acquisition voxel = 1.875 mm × 1.875 mm × 4 mm; slice number = 36; number of signal average = 1; labeling duration = 1,500 ms; postlabeling delay time = 2,000 ms; acquisition time = 3 min 34 s. Additionally, axial 3 mm FLAIR images were acquired to assess WM lesions.

### 2.3 Image preprocessing

ASL image preprocessing was carried out using the ASAP (Automatic Software for ASL Processing) toolbox (Mato, García‐Polo, O'Daly, Hernández‐Tamames, & Zelaya, 2016). First, the T1w structural and ASL images were reoriented to the anterior commissure–posterior commissure (AC–PC) plane, and the image origin was set to the AC. Second, structural images were segmented into GM, WM, and CSF. Thus, the segmentation of GM and WM probability maps were obtained, as well as the brain mask for the brain subtraction of ASL images. Third, the structural images were co‐registered to ASL images and downsampled into the ASL space. Fourth, partial volume correction (PVC) was performed using Asllani estimates in ASL image space, which could estimate the CBF for GM and WM independently (Mato et al., 2016). Then, the subtracted and PVC ASL images within the brain mask were normalized into the MNI space (voxel values were interpolated and upsampled into 2 mm × 2 mm × 2 mm) based on the co‐registration and segmentation nonlinear transformation information. Finally, the corrected CBF images of GM and WM were smoothed with a 3D Gaussian kernel (full‐width at half maximum, FWHM = 6 mm × 6 mm × 6 mm).

For whole brain (WB) level comparisons, the mean CBF within GM, WM, and WB (GM + WM) were calculated. A CBF ratio of GM/WM was obtained by dividing the mean CBF within GM by the mean CBF within WM. For voxel level comparisons, voxel‐based analysis (VBA) was performed to find the altered CBF of the local brain regions.

### 2.4 Statistical analysis

Statistical analyses were carried out using IBM SPSS statistics Version 25 and the DPABI toolbox (a toolbox for Data Processing & Analysis of Brain Imaging. Release V3.1; Yan, Wang, Zuo, & Zang, 2016). For measurement data, parametric ANOVA or nonparametric Kruskal–Wallis H analysis was carried out and followed by corresponding post hoc two‐sample tests (Tukey's test for parametric analysis and the Mann–Whitney *U* test for nonparametric analysis). For categorical data, Fisher's exact test was carried out. For ASL images, voxel‐based statistical analysis was performed using ANOVA with age as a covariate, followed by a pairwise two‐sample *t*‐test with 5,000 permutations to define the t‐value threshold. An uncorrected *p* < .01 and cluster size >30 voxels were considered to be a significant difference. The Pearson correlation coefficient or the Spearman rank correlation, with age as a covariate, was applied to find the relationship between the values extracted from brain regions, WM lesions and the clinical evaluations (SLEDAI, SLICC, disease duration, and the total cumulative duration of steroid use). For the above analysis, a *p* < .05 was considered a significant difference. Receiver operating characteristic (ROC) analysis was carried out to differentiate NPSLE from non‐NPSLE by the CBF of the WB level or voxel level, which showed significant differences between NPSLE and non‐NPSLE. The cut‐off value was determined according to the maximum Youden index.

### 2.5 Support vector machine learning

Support vector machine (SVM) with a Gaussian kernel function based on LIBSVM (Library for Support Vector Machines) and MATLAB scripts (MATLAB 2014b) was adopted for the classification of NPSLE and non‐NPSLE patients and NCs (Chang & Lin, 2011). The mean CBF values extracted from the brain regions according to voxel level statistical results were used as features for the classification. Leave‐one‐out cross validation (LOOCV) was applied to SVM training and predictions. The accuracy, sensitivity, specificity, precision, recall, and F1‐score were used for the SVM performance evaluation.

## 3 RESULTS

### 3.1 General demographics and clinical characteristics

The subjects were all females. There was no significant difference in age between NPSLE and non‐NPSLE patients and NCs. Significant differences were identified between NPSLE and non‐NPSLE patients for SLEDAI and SLICC scores but not for disease duration, total cumulative duration of steroid use or WM lesion volumes (for a WM lesion distribution map, see Figure S1). NPSLE patients presented different clinical neurological symptoms including seizures or epilepsy (14, 45.2%), cognitive disorders (12, 38.7%), mood disorders (9, 29.0%), neuromyelitis optica spectrum disorders (NMOSD) (9, 29.0%), headache (8, 25.8%), large vessel stroke (6, 19.4%), psychosis (6, 19.4%), and acute disturbances of consciousness (5, 16.1%). For more clinical information, refer to Table 1 and Table S1.

### 3.2 WM lesions

Twenty‐three NPSLE and six non‐NPSLE patients showed WM lesions on FLAIR images. There was a significant difference in the incidence of WM lesions between NPSLE and non‐NPSLE patients (*p* < .001, refer to Table S1), but there was no significant difference in WM lesion volumes (*p* = .469). As the WM lesion distribution map (Figure S1) shows, the WM lesions were mainly distributed within the periventricular and deep WM.

### 3.3 Perfusion abnormalities

The WB level results are listed in Table 1. The ratio of GM/WM showed a decreasing pattern for NC–non‐NPSLE–NPSLE. Significant differences were found for NCs versus non‐NPSLE patients and for non‐NPSLE versus NPSLE patients in the CBF ratio of GM/WM.

**Figure 1:**
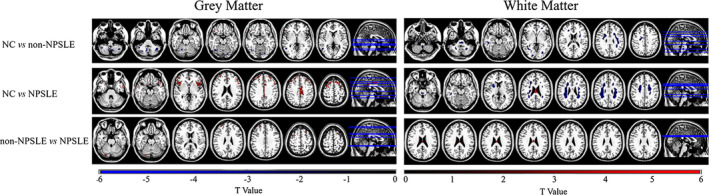
VBA results (*t*‐value distribution) of GM and WM for NCs versus non‐NPSLE, NCs versus NPSLE, and non‐NPSLE versus NPSLE (two‐sample *t*‐test with 5,000 permutations to define the *t*‐value threshold. An uncorrected *p* < .01 and cluster size>30 voxels were considered to indicate a significant difference. The color bar shows the range of the *t*‐values displayed in the figures

VBA was performed for WM and GM separately. Figure 1 shows the results of WM and GM for non‐NPSLE versus NPSLE patients, NCs versus non‐NPSLE patients and NCs versus NPSLE patients, respectively. The details are presented in Table 2.

**Table 2:** The brain regions with significantly different CBFs among NPSLE and non‐NPSLE patients and NCs, and the correlation with clinical variables

Between groups	GM or WM	Cluster index	Brain regions	No. of voxels	Peak *t* value	MNI coordinates (mm)			Correlation with SLEDAI	Correlation with SLICC
						*X*	*Y*	*Z*		
Non‐NPSLE–NPSLE	GM	1	Right cerebellum posterior lobe	41	3.05	26	−84	−24	–	−0.33^a^ (0.015)
		2	Left inferior/middle frontal gyrus	31	3.50	−46	44	14	–	–
		3	Right superior frontal gyrus	49	3.72	30	50	32	–	−0.28^a^ (0.044)
		4	Right middle frontal gyrus	37	3.63	36	6	58	–	−0.38^a^ (0.005)
		5	Left superior frontal gyrus	45	3.59	−20	20	60	–	−0.29^a^ (0.034)
	WM	1	Corpus callosum	34	3.28	0	−16	26	–	−0.32^a^ (0.017)
NC–Non‐NPSLE	GM	1	Part of right cerebellum posterior lobe	137	−3.03	8	−62	−28	–	–
		2	Part of left cerebellum posterior lobe	144	−2.99	−42	−60	−42	–	–
		3	Part of right cerebellum posterior lobe	235	−2.85	40	−68	−36	–	–
		4	Part of right cerebellum anterior/posterior lobe	99	−3.37	32	−48	−36	–	–
		5	Part of left cerebellum anterior lobe	41	−2.91	−30	−52	−34	–	–
		6	Part of left cerebellum posterior lobe	136	−3.00	−6	−64	−36	–	–
		7	Right inferior/middle temporal gyrus	39	−2.69	56	−42	−12	−0.27^b^ (0.045)	–
		8	Part of right cerebellum posterior lobe	40	−2.66	20	−60	−20	–	–
		9	Left lingual gyrus	42	−3.08	−22	−82	−14	−0.35^b^ (0.010)	–
		10	Right inferior frontal gyrus	77	3.73	56	30	−2	–	–
		11	Right middle temporal gyrus	85	−3.37	50	−54	4	−0.29^b^ (0.035)	–
	WM	1	Brainstem (right and left)	38	−2.74	2	−42	−52	–	–
		2	Left cerebellum anterior/posterior lobe	144	−3.41	−26	−60	−42	–	–
		3	Left and right brainstem (pons)	318	−3.19	−6	−28	−44	–	–
		4	Right cerebellum anterior/posterior lobe	305	−3.19	22	−60	−34	–	–
		5	Left inferior temporal lobe	30	−2.43	−50	−32	−20	–	–
		6	Right inferior/middle temporal lobe; right middle occipital lobe	420	−3.06	52	−26	−20	–	–
		7	Left lingual lobe	53	−3.56	−14	−88	−10	–	–
		8	Right lingual/calcarine/cuneus	169	−4.00	18	−92	−6	–	–
		9	Left (middle) occipital lobe	73	−3.23	−26	−78	−4	–	–
		10	Left sub‐gyral (frontal lobe)	119	−2.65	−26	42	0	–	–
		11	Right sub‐gyral (frontal lobe)	143	−2.41	24	34	18	–	–
		12	Left precentral; inferior parietal gyrus	554	−3.13	−42	−28	30	–	–
		13	Right cingulate/precentral lobe	576	−2.67	24	−20	56	−0.41^b^(0.002)	–
		14	Left inferior parietal lobe	74	−2.53	−32	−40	30	–	–
		15	Left sub‐gyral (frontal lobe)	47	−2.35	−28	−32	34	–	––
NC–NPSLE	GM	1	Left superior/middle temporal	251	4.68	−54	6	−34	–	–
		2	Right superior temporal gyrus	104	3.95	56	14	−10	–	–
		3	Right middle temporal gyrus	68	3.30	58	6	−26	–	–
		4	Right rectal gyrus (frontal lobe)	51	4.36	4	14	−24	–	–
		5	Left inferior/middle frontal gyrus; insula; precentral gyrus	882	5.56	−48	40	16	–	–
		6	Right superior/inferior/middle frontal gyrus; insula; supplementary motor area	1,484	5.11	30	50	30	–	–
		7	Left medial frontal gyrus; left anterior cingulum gyrus	160	5.17	−2	42	22	–	–
		8	Right medial frontal gyrus	52	3.59	4	38	30	–	–
		9	Left superior/middle frontal gyrus; left precentral gyrus	654	5.44	−28	28	50	–	−0.21^a^ (0.045)
		10	Bilateral middle cingulum gyrus	555	4.54	0	−6	40	–	–
	WM	1	Pons	76	−2.80	4	−28	−28	–	–
		2	Right temporal lobe	55	−3.09	52	−26	−22	–	–
		3	Right frontal/parietal lobe; limbic lobe; cingulate gyrus; putamen;	1,799	−3.24	22	4	8	–	–
		4	Corpus callosum	108	5.36	0	−16	26	–	−0.30^a^ (0.026)
		5	Left frontal/ parietal lobe; cingulate gyrus	888	−3.22	−28	−46	28	−0.28^b^ (0.038)	–

*Note*:The correlation with clinical variable was presented by R(P).

a Spearman rank correlation.

b Pearson's correlation.

Compared to NCs, NPSLE patients demonstrated decreased GM CBF in the bilateral superior and middle temporal gyrus, parts of the frontal gyrus and bilateral middle cingulum gyrus. Increased CBF was seen in WM located in the pons, right temporal lobe, bilateral frontal lobe, bilateral partial lobe, and bilateral limbic lobe, and decreased CBF was seen in the corpus callosum in NPSLE patients.

Non‐NPSLE patients showed increased GM CBF predominantly in the bilateral cerebellum, right temporal gyrus, and left lingual gyrus and showed decreased CBF in the right inferior frontal gyrus compared to NCs. Distributed WM showed increased CBF, which included the WM located in the brainstem, bilateral cerebellum lobes, inferior temporal lobe, right middle temporal lobe, bilateral middle occipital lobe, left lingual/calcarine/cuneus, left inferior partial lobe, right cingulate/precentral lobe, and parts of the frontal lobe.

Compared to non‐NPSLE patients, decreased GM CBF was found in the right cerebellum posterior lobe, left inferior frontal gyrus, bilateral superior frontal gyrus, and bilateral middle frontal gyrus, while decreased WM CBF was found in the corpus callosum in NPSLE patients.

### 3.4 Relationship between perfusion measures, clinical variables, and WM lesion volume

Abnormal CBF within several brain regions (e.g., cingulate, corpus callosum, and bilateral frontal gyrus) showed weak correlations with clinical information including SLEDAI and SLICC scores and showed no significant correlation with disease duration, total cumulative duration of steroid use, or WM lesion volume when age was used as a covariate. The details of this analysis are shown in Table 2.

We found no significant association between CBF measures and other clinical information (e.g., renal disorder) or laboratory parameters (e.g., anti‐dsDNA) related to the manifestation of SLE, vascular risk factors and immunological indicators.

### 3.5 ROC analysis to differentiate NPSLE from non‐NPSLE

Differentiating non‐NPSLE and NPSLE is a challenge in clinical practice. In this work, the ratio of GM/WM was used to distinguish non‐NPSLE from NPSLE. The ROC analysis results are shown in Table 3 and Figure S2. The GM/WM ratio has a moderate ability to distinguish NPSLE from non‐NPSLE, with an AUC of 0.737 and a cut‐off value of 1.96 (sensitivity = 83.3%, specificity = 64.5%, and Youden index = 0.478).

**Table 3:** Details of ROC analysis for differentiating NPSLE from non‐NPSLE by the CBF ratio of GM/WM and CBF within the local brain regions detected by VBA

	AUC	Cut‐off value	Youden index	Sensitivity (%)	Specificity (%)
Ratio of GM/WM	0.737	1.96	0.478	83.3	64.5
Right cerebellum posterior lobe	0.742	44.04	0.381	83.3	54.8
Left inferior/middle frontal Gyrus	0.743	38.54	0.446	83.3	61.3
Right superior frontal Gyrus	0.738	32.34	0.492	75.0	74.2
Right middle frontal Gyrus	0.780	26.03	0.492	75.0	74.2
Left superior frontal Gyrus	0.770	27.45	0.441	66.7	77.4
Corpus callosum	0.738	17.08	0.433	91.7	51.6

**Figure 2:**
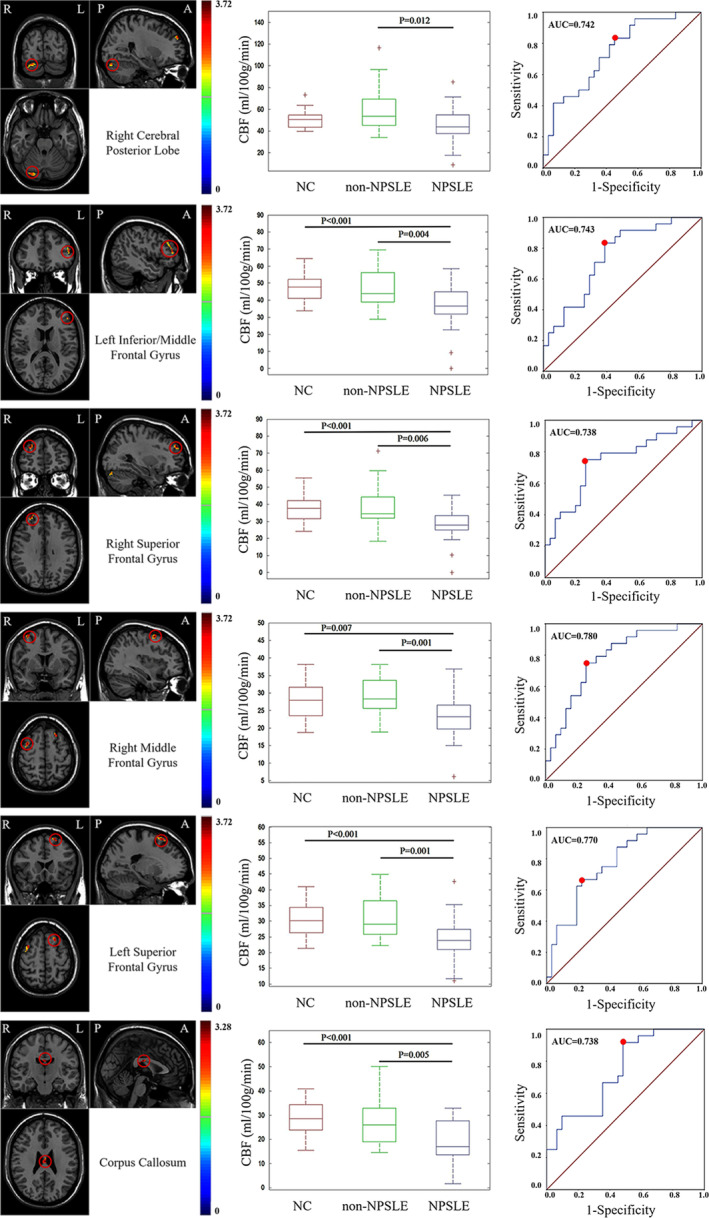
ROC analysis of CBF extracted from statistically significant brain regions to differentiate NPSLE and non‐NPSLE. The red point indicates the cut‐off value

We also investigated the ability of regional brain measurements to differentiate NPSLE from non‐NPSLE. ROC analysis showed that all the statistically significant brain regions, such as the right cerebellum posterior lobe, bilateral superior and middle frontal gyrus and corpus callosum, showed a moderate performance (AUC > 0.7) for differentiating non‐NPSLE from NPSLE. The details of this analysis are shown in Table 3 and Figure 2.

### 3.6 Classification by SVM

The classification results are listed in Table 4. CBF in the corpus callosum can distinguish non‐NPSLE from NPSLE with high accuracy (83.6%), sensitivity (80.6%), and specificity (87.5%) and is superior to the method using brain regions within GM. For NCs versus non‐NPSLE and NCs versus NPSLE, the combined features derived from GM and WM showed good classification. For NCs versus non‐NPSLE + NPSLE, the classification results were good (accuracy of 87.4%, sensitivity of 89.1%, and specificity of 84.4%); however, differentiating NCs + non‐NPSLE from NPSLE showed low sensitivity (61.3%).

**Table 4:** SVM classification results for different groups

Classification groups	Features (No.)	Accuracy (%)	Sensitivity (%)	Specificity (%)	Precision (%)	Recall (%)	F1‐socre
non‐NPSLE versus NPSLE	GM (5)	72.7	71.0	75.0	78.6	71.0	0.746
	WM (1)	83.6	80.6	87.5	89.3	80.6	0.847
	GM + WM (6)	83.6	80.6	87.5	89.3	80.6	0.847
NC versus non‐NPSLE	GM (11)	80.4	70.8	87.5	81.0	70.8	0.756
	WM (15)	76.8	50.0	96.7	92.3	50.0	0.649
	GM + WM (26)	83.9	70.8	93.8	89.5	70.8	0.791
NC versus NPSLE	GM (10)	76.2	80.6	71.9	73.5	80.6	0.769
	WM (5)	82.5	71.0	93.8	91.7	71.0	0.800
	GM + WM (15)	84.1	77.4	90.6	88.9	77.4	0.828
NC versus non‐NPSLE + NPSLE	GM (26)	86.2	92.7	75.0	86.4	92.7	0.895
	WM (21)	81.6	94.5	59.4	80.0	94.5	0.867
	GM + WM (47)	87.4	89.1	84.4	90.7	89.1	0.899
NC + non‐NPSLE versus NPSLE	GM (26)	79.3	58.1	91.1	78.3	58.1	0.667
	WM (21)	81.6	61.3	92.9	82.6	61.3	0.704
	GM + WM (47)	82.8	61.3	94.6	86.4	61.3	0.717

## 4 DISCUSSION

In the present study, we identified different cerebral perfusion patterns between non‐NPSLE and NPSLE patients. In non‐NPSLE patients, within GM, CBF was decreased in the right inferior frontal gyrus but increased in the bilateral cerebellum, right temporal gyrus and left lingual gyrus compared to NCs. Within WM, distributed increased CBF was observed compared to NCs. In NPSLE patients, CBF in the bilateral frontal gyrus, temporal gyrus and middle cingulum gyrus decreased compared to that in NCs, while CBF was increased in most of the WM regions except the corpus callosum. The main differences between NPSLE and non‐NPSLE were in the frontal lobe and cerebellum (GM) and in the corpus callosum (WM). Perfusion patterns can accurately classify NPSLE and non‐NPSLE patients with CBF, which could be a potential imaging biomarker for the differentiation of non‐NPSLE and NPSLE.

Our data demonstrated an increase in GM perfusion in non‐NPSLE patients and a decrease in NPSLE patients, implying the dynamic progression of perfusion alterations. Cohen et al. (2017) proposed that vascular injury may occur in all patients with SLE, but primary NP manifestations occurred only after exceeding a certain threshold of ischemic injury, which is consistent with our findings. CBF significantly increased in GM in the temporal gyrus, cingulate gyrus, and cerebellum regions and in widespread WM in non‐NPSLE patients compared with NCs. We hypothesized that this result may be caused by a compensatory mechanism in response to ischemia or injury, which was consistent with previous fMRI studies showing hyperactivities and hyperconnectivity within certain specific networks (e.g., the sensorimotor network) (Mikdashi, 2016; Niu et al., 2018; Nystedt et al., 2018; Papadaki et al., 2014; Wu et al., 2018; Zhang et al., 2017). However, in NPSLE patients, along with widespread vasculopathy and thrombosis, the perfusion of these brain regions was significantly decreased, which means a large reduction in compensatory capacity when the damage exceeded the threshold. In this work, 18 NPSLE patients showed obvious cerebral atrophy (only four non‐NPSLE patients showed cerebral atrophy), which could also account for GM hypoperfusion. Non‐NPSLE and NPSLE might share a similar vessel compensatory mechanism within WM, which presented in higher perfusion in distributed areas within WM both in non‐NPSLE and NPSLE patients. This finding is consistent with a previous DSC study showing elevated CBF and CBV within normal‐appearing WM in SLE patients compared to that in NCs (Gasparovic et al., 2010). The hyperpefusion within WM might also be attributed to potential vascular dysfunction caused by evaluated blood pressure in SLE patients (Gasparovic, Qualls, Greene, Sibbitt, & Roldan, 2012).

Histopathological studies revealed that nonspecific focal vasculopathy appeared in non‐NPSLE, while diffuse vasculopathy and microthrombi were commonly found in NPSLE, which is the possible pathological basis for different patterns of perfusion abnormalities (Cohen et al., 2017; Sarbu, Sarbu, Bargallo, & Cervera, 2018; Sibbitt et al., 2010). Because of its vasculopathy nature, NPSLE is considered a subtype of inflammatory and immunologically mediated small vessel diseases. The corpus callosum is a key WM structure connecting hemisphere projection fibers. This territory is prone to be affected by hemodynamic impairment. Previous work found a decreased NAA/Cr ration or an increased Cho/Cr ratio within the posterior cingulate cortex and posterior region of the corpus callosum, especially in hypoperfused areas (Castellino et al., 2005; Wang et al., 2012). Additionally, the corpus callosum was the region most commonly reported to have abnormal diffusion parameters (including fractional anisotropy [FA] and mean diffusivity [MD]). Together with our finding of abnormal perfusion in the corpus callosum, these findings indicated that the corpus callosum was one of the fibers specifically damaged in SLE patients. This damage was more pronounced in NPSLE patients, indicating that the corpus callosum measurement could be a sensitive biomarker for defining NPSLE (Costallat et al., 2018; Lee et al., 2015; Wang et al., 2012). This perfusion pattern in the corpus callosum needs to be validated in a large sample with a longitudinal design and has the potential to be an imaging biomarker for defining SLE patients vulnerable to progress to NPSLE.

Some previous works showed conflicting results demonstrating the hypoperfusion of GM and WM in both NPSLE and non‐NPSLE patients (Papadaki et al., 2018; Wang et al., 2012). This discordant information may be due to variations in the sample sizes, MR scanners or analysis methods of these studies (Emmer et al., 2010; Wang et al., 2012; Zimny et al., 2014). For example, previous works performed CBF normalization with the assumption of stable perfusion within some brain regions (such as the cerebellum). However, we found that the perfusion within the cerebellum showed a significant difference between NPSLE and non‐NPSLE patients, indicating that CBF in the cerebellum was altered by SLE disease. Therefore, we did not normalize cerebral CBF with CBF in the cerebellum. The alterations in cerebellum CBF and their relationship with cerebral perfusion should be studied in future studies.

In the current study, CBF in the right cerebellum, posterior lobe, right middle frontal gyrus, and corpus callosum were correlated with SLICC measures in NPSLE patients, suggesting that the hypoperfusion of certain cerebral regions could be an imaging biomarker for predicting prognosis. A correlation was observed between CBF in the left lingual gyrus (GM), right cingulate/precentral lobe (WM), and SLEDAI measures in non‐NPSLE patients, implying that the hypoperfusion of these brain areas could reflect high disease activity that requires aggressive treatment.

The differentiation of NPSLE patients from non‐NPSLE patients and NCs is essential in clinical practice. The previously proposed imaging measures have high sensitivity (80–100%) but relatively low specificity (<67%) with PET, SPECT or DSC‐MRI (Cervera et al., 2006; Emmer et al., 2010; Kao, Ho, et al., 1999; Kao, Lan, et al., 1999). In the present study, we found that the differentiation of NPSLE patients from non‐NPSLE patients could reach a high sensitivity and specificity of 71.0 and 75.0%, respectively, for GM features; 83.6 and 80.6%, respectively, for WM features; and 83.6 and 80.6%, respectively, for combined GM and WM features. Using a simple cut‐off value of 1.96 for the GM/WM CBF ratio, we can differentiate NPSLE and non‐NPSLE patients with a sensitivity of 83.3% and a specificity of 64.5%. In particular, CBF in the corpus callosum showed good differential performance using SVM, implying that this measure could be a noninvasive and objective imaging biomarker for the early accurate diagnosis of NPSLE, especially for subclinical and atypical NPSLE.

There are some limitations to this study. First, this study is a cross‐sectional single MRI modality (perfusion) study with a relatively small sample size from a single center. A longitudinal study using multimodality techniques, such as structural and functional MRI and PET/SPECT, in a large sample size from multi‐site centers is warranted to validate the current findings. Second, cognitive and neuropsychiatric assessments were not comprehensively performed, hindering the investigation between perfusion and neuropsychiatric scores. Third, the lesion effect on CBF alterations in SLE is not fully demonstrated in the current study. Further study with longitudinal data and comprehensive analysis is required to investigate the association between lesions and CBF in SLE patients (Gasparovic et al., 2010). Fourth, SVM analysis was performed according to the current state‐of‐the art approach using a cross‐validation approach, which needs further validation in larger multi‐site studies in a clinical scenario (Haller, Lovblad, Giannakopoulos, & Van De Ville, 2014).

## 5 CONCLUSION

Different brain perfusion patterns were observed in NPSLE and non‐NPSLE patients using the noninvasive ASL technique and were correlated with clinical scores, implying that perfusion characteristics could be a useful biomarker reflecting pathophysiological alterations and identifying NPSLE.

## CONFLICT OF INTEREST

None declared.

## AUTHOR CONTRIBUTIONS

The project was designed, conceived and planned by Y.L., X.Z., and H.L. The data acquisition was performed by L.S. The data were processed and analyzed by Z.Z. The article was written and edited by Z.Z. and L.S. The article was revised by Y.D., J.H., X.Q., and S.H. All authors approved the final version of the manuscript.

## PATIENT CONSENT FOR PUBLICATION

Obtained.

## Supporting information


**Table S1** Details of the clinical information and laboratory parameters of the SLE subjects included in this work.

**Figure S1** The white matter lesion probability map. The map was presented by the ratio of the number of SLE patients with lesions and the total number of SLE patients as shown in the color bar.

**Figure S2** The ROC analysis of GM/WM ratio for the differentiation of NPSLE and non‐NPSLE. The red point indicated the cut‐off value.

## Data Availability

The data that support the findings in this study are available from the corresponding author upon request.
