# Supplementary material for: Different patterns of cerebral perfusion in SLE patients with and without neuropsychiatric manifestations
Source: Hum Brain Mapp. 2019 Oct 24;41(3):755–66. doi: 10.1002/hbm.24837 (PMC7268026; doi:10.1002/hbm.24837)
Supplement: Supplementary file 1 — Table S1 Details of the clinical information and laboratory parameters of the SLE subjects included in this work. [file HBM-41-755-s002.docx]

Supplementary Table 1. Details of the clinical information and laboratory parameters of the SLE subjects included in this work.

| Parameters | NPSLE  n=31 | nonNPSLE  n=24 | P |
| --- | --- | --- | --- |
| ACR/SLICC clinical manifestations |  |  |  |
| Typical rash, n (%) | 23(74.2) | 15(62.5) | 0.014^*^ |
| Alopecia, n (%) | 15(48.4) | 12(50.0) | 0.906 |
| Oral ulcers, n (%) | 8(25.8) | 8(33.3) | 0.542 |
| [Raynaud's phenomenon](javascript:;), n (%) | 4(12.9) | 4(16.7) | 0.695 |
| Cutaneous vasculitis, n (%) | 6(19.4) | 6(25.0) | 0.615 |
| Non-erosive arthritis, n (%) | 15(48.4) | 12(50.0) | 0.906 |
| Serositis, n (%) | 11(35.5) | 6(25.0) | 0.404 |
| Renal disorder, n (%) | 19(61.4) | 12(50.0) | 0.402 |
| Active cases, n (%) | 9(29.0) | 4(16.7) | 0.284 |
| Hematologic abnormalities, n (%) | 18(58.1) | 18(75.0) | 0.190 |
| Leukopenia, n (%) | 15(48.4) | 12(50.0) | 0.906 |
| Anemia, n (%) | 13(41.9) | 9(37.5) | 0.739 |
| Thrombocytopenia, n (%) | 6(19.4) | 3(12.5) | 0.496 |
| Interstitial pneumonia, n (%) | 6(19.4) | 2(8.3) | 0.250 |
| Cardiac involvement n (%) | 4(12.9) | —— |  |
| Myalgia, n (%) | 3(9.7) | 6(25.0) | 0.128 |
| Digestive system involvement, n (%) | 2(6.5) | 1(4.2) | 0.711 |
| Serologic abnormalities |  |  |  |
| Anti-dsDNA positive, n (%) | 19(61.3) | 12(50.0) | 0.402 |
| Anti-Sm positive, n (%) | 9(29.0) | 14(58.3) | 0.029 |
| Antiphospholipid antibodies positive, n(%) | 12(38.7) | 5(20.8) | 0.155 |
| Anti-RNP, n (%) | 15(48.4) | 15(62.5) | 0.297 |
| Anti-ribosomal-P protein, n (%) | 16(51.6) | 8(33.3) | 0.175 |
| Anti-Ro/SSA, n (%) | 23(74.2) | 19(79.2) | 0.667 |
| Anti-La/SSB, n (%) | 5(16.1) | 6(25.0) | 0.415 |
| AHA, n (%) | 5(16.1) | 6(25.0) | 0.415 |
| ANuA, n (%) | 8(25.8) | 6(25.0) | 0.946 |
| Low levels of complements, n (%) | 29(93.6) | 19(79.2) | 0.113 |
| Specific & generic risk factors |  |  |  |
| Sjogren’s syndrome, n (%) | 7(22.6) | 2(8.3) | 0.157 |
| Hashimoto thyroiditis, n (%) | 5(16.1) | 1(4.2) | 0.158 |
| Hypertension history, n (%) | 8(25.8) | 1(4.2) | 0.031^*^ |
| Diabetes, n (%) | 1(3.2) | 1(4.2) | 0.853 |
| Dyslipidemia, n (%) | 3(9.7) | —— |  |
| Low levels of WBC, n (%) | 15(48.4) | 12(50.0) | 0.906 |
| Low levels of Hb, n (%) | 13(41.9) | 9(37.5) | 0.739 |
| Low levels of PLT, n (%) | 6(19.4) | 3(12.5) | 0.496 |
| NP, n（%） |  |  |  |
| Seizures or epilepsy, n (%) | 14(45.2) | —— |  |
| Psychosis, n (%) | 6(19.4) | —— |  |
| Mood disorders, n (%) | 9(29.0) | —— |  |
| Cognitive disorders, n (%) | 12(38.7) | —— |  |
| NMOSD, n (%) | 9(29.0) | —— |  |
| Large vessel stroke, n (%) | 6(19.4) | —— |  |
| Headache, n (%) | 8(25.8) | —— |  |
| Acute disturbance of consciousness, n(%) | 5(16.1) | —— |  |
| Diffused NP | 22(72.0) |  |  |
| Localized NP | 10(32.3) |  |  |
| Active NP, n (%) | 11(35.5) | —— |  |
| NP as the first symptom of SLE, n (%) | 11(35.5) | —— |  |
| MRI features at the study scan |  |  |  |
| WM lesions, n(%) | 23(74.2) | 6(25.0) | <0.001^*^ |
| Cerebral atrophy, n(%) | 18(58.1) | 4(16.7) | 0.002 |

Note: * indicate there is statistical difference between groups based on chi-square test.
