# Supplementary material for: Different patterns of cerebral perfusion in SLE patients with and without neuropsychiatric manifestations
Source: Hum Brain Mapp. 2019 Oct 24;41(3):755–66. doi: 10.1002/hbm.24837 (PMC7268026; doi:10.1002/hbm.24837)
Supplement: Supplementary file 2 — Figure S1 The white matter lesion probability map. The map was presented by the ratio of the number of SLE patients with lesions and the total number of SLE patients as shown in the color bar. [file HBM-41-755-s001.docx]

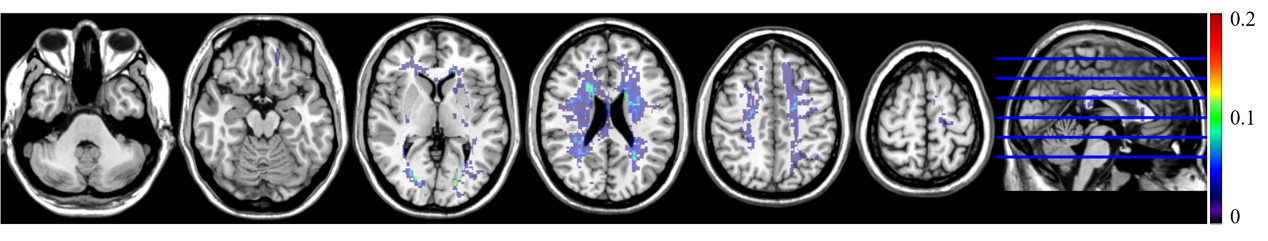


Supplementary Figure 1. The white matter lesion probability map. The map was presented by the ratio of the number of SLE patients with lesions and the total number of SLE patients as shown in the color bar.
