# Supplementary material for: Different patterns of cerebral perfusion in SLE patients with and without neuropsychiatric manifestations
Source: Hum Brain Mapp. 2019 Oct 24;41(3):755–66. doi: 10.1002/hbm.24837 (PMC7268026; doi:10.1002/hbm.24837)
Supplement: Supplementary file 3 — Figure S2 The ROC analysis of GM/WM ratio for the differentiation of NPSLE and non‐NPSLE. The red point indicated the cut‐off value. [file HBM-41-755-s003.docx]

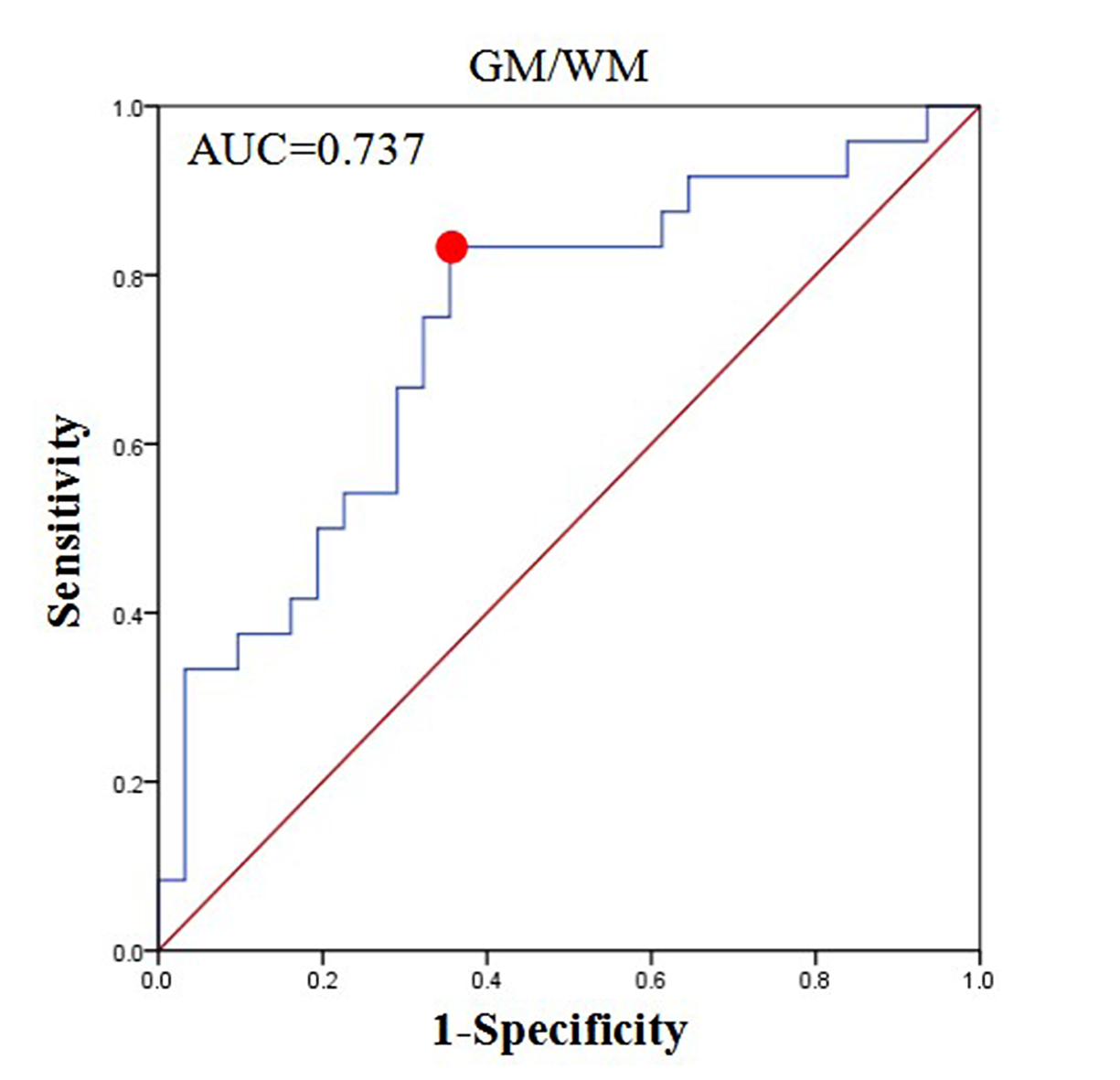


Supplementary Figure 2. The ROC analysis of GM/WM ratio for the differentiation of NPSLE and nonNPSLE. The red point indicated the cut-off value.
